# An analysis by metabolic labelling of the encephalomyocarditis virus ribosomal frameshifting efficiency and stimulators

**DOI:** 10.1099/jgv.0.000888

**Published:** 2017-08-08

**Authors:** Roger Ling, Andrew E. Firth

**Affiliations:** Division of Virology, Department of Pathology, University of Cambridge, Cambridge CB2 1QP, UK

**Keywords:** gene expression, protein synthesis, translational control, ribosomal frameshifting, genetic recoding, virus

## Abstract

Programmed −1 ribosomal frameshifting is a mechanism of gene expression whereby specific signals within messenger RNAs direct a proportion of ribosomes to shift −1 nt and continue translating in the new reading frame. Such frameshifting normally depends on an RNA structure stimulator 3′-adjacent to a ‘slippery’ heptanucleotide shift site sequence. Recently we identified an unusual frameshifting mechanism in encephalomyocarditis virus, where the stimulator involves a *trans*-acting virus protein. Thus, in contrast to other examples of −1 frameshifting, the efficiency of frameshifting in encephalomyocarditis virus is best studied in the context of virus infection. Here we use metabolic labelling to analyse the frameshifting efficiency of wild-type and mutant viruses. Confirming previous results, frameshifting depends on a G_GUU_UUU shift site sequence and a 3′-adjacent stem-loop structure, but is not appreciably affected by the ‘StopGo’ sequence present ~30 nt upstream. At late timepoints, frameshifting was estimated to be 46–76 % efficient.

## Abbreviations

EMCV, encephalomyocarditis virus; IRES, internal ribosome entry site; p.i., post infection; PRF, programmed ribosomal frameshifting; PTC, premature termination codon; TMEV, Theiler's murine encephalomyelitis virus.

## Full-Text

Programmed −1 ribosomal frameshifting (−1 PRF) is utilized in the expression of many viral genes and some cellular genes [1]. Sites of −1 PRF generally comprise a ‘slippery’ sequence (at which the change in reading frame occurs) and a 3′-adjacent stimulatory mRNA structure [2]. In eukaryotes, the slippery sequence fits a consensus motif X_XXY_YYZ, where XXX is any three identical nucleotides (although certain exceptions occur, such as GGU); YYY represents AAA or UUU; Z represents A, C or U; and underscores separate zero-frame codons. The stimulatory mRNA structure generally comprises a stem-loop or a pseudoknot and is nearly always separated from the shift site by a ‘spacer’ region of 5–9 nt. Typical −1 PRF efficiencies fall in the range 5–50 %.

**Fig. 1.**
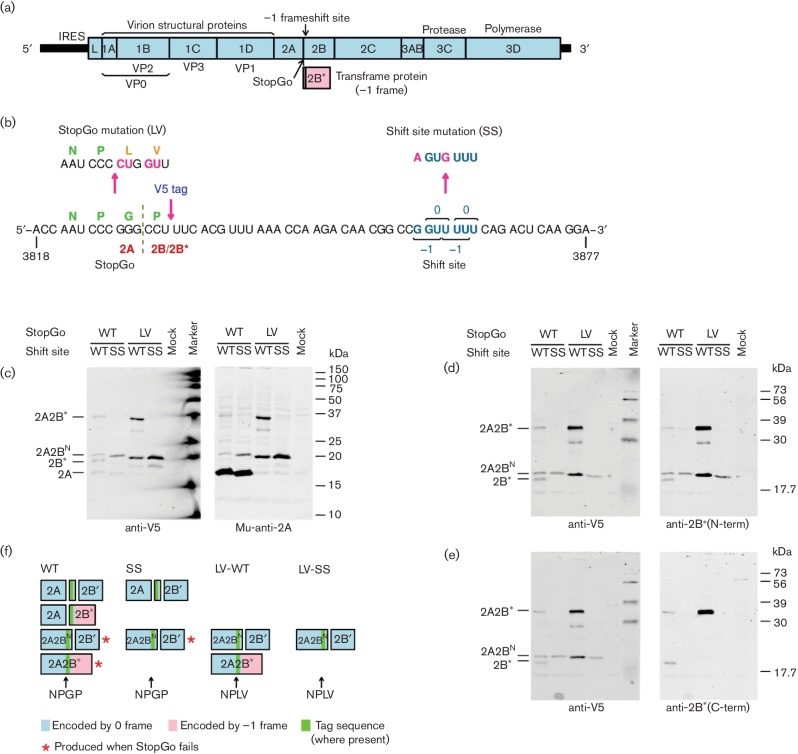
Analysis of 2A and 2B* expression in V5-tagged wild-type (WT) and mutant EMCVs. (a) Map of the ~7700 nt EMCV genome. The 5′ and 3′ UTRs are indicated in black and the polyprotein ORF is indicated in pale blue with subdivisions showing mature cleavage products. The overlapping 2B* ORF is indicated in pale pink. (b) Mutations introduced to prevent PRF (SS), and StopGo-mediated co-translational separation at the C-terminus of 2A (LV). Additional mutants were constructed with sequence encoding a V5 tag. (c–e) Western blot analysis of virus-infected cell lysates. BHK-21 (c) or L929 (d, e) cells were infected with V5-tagged WT or mutant viruses at m.o.i.~10 and lysates prepared at 8 h post infecton (p.i.). Anti-V5 antibodies were from rabbit (c) or mouse (d, e) along with the appropriate secondary antibody labelled with IRDye 680. Antibodies Mu-anti-2A, anti-2B*(N-term) and anti-2B*(C-term) were used with the appropriate secondary antibody labelled with IRDye 800. Left and right panels show scans from, respectively, the 700 and 800 nm channel of images obtained with a LiCor Odyssey scanner. Different markers were used for (c) and (d, e) with some differences between the two. (f) Schematic summary of 2A-, 2B- and 2B*-related products for WT and mutant EMCVs.

Like other members of the family *Picornaviridae*, encephalomyocarditis virus (EMCV) has a single-stranded RNA genome of positive polarity which also serves as an mRNA. Translation initiation is mediated by an internal ribosome entry site (IRES) within the 5′ UTR. Translation of a single long ORF produces a polyprotein that is proteolytically cleaved, mainly by the virus-encoded 3C protease (Fig. 1a). Separation between 2A and 2B, however, occurs co-translationally via a mechanism known as ‘StopGo’ or ‘Stop–Carry On’ that depends critically on the amino acid motif D(V/I)ExNPGP (where the last proline is the first amino acid of 2B) [3, 4]. A −1 PRF site is present in EMCV just downstream of the junction between the 2A- and 2B-encoding regions of the polyprotein ORF. When PRF occurs, ribosomes translate the transframe fusion protein, 2B*, comprising the N-terminal 12 amino acids of 2B together with 117 C-terminal amino acids encoded within the −1 frame (Fig. 1a) [5]. Frameshifting in EMCV is thought to be important both to express the 2B* protein (whose function is currently unknown) and to downregulate expression of the viral enzymatic proteins [5–7]. The PRF mechanism in EMCV is atypical due to the apparent absence of an appropriately spaced stimulatory RNA structure, and an initial failure to reconstitute PRF outside of the context of virus infection [5]. Thus, in contrast to other examples of −1 frameshifting which can be studied using exogeneous reporter constructs, it was informative to assess the efficiency and mechanism of frameshifting in the context of the virus genome during virus infection.

Using ribosome profiling of virus-infected cells, we recently discovered that the frameshifting efficiency in EMCV varies over the course of infection from negligible levels [at 2 h post infection (p.i.)] to 70 % (at 8 h p.i.), and so, by diverting the bulk of ribosomes out of the polyprotein ORF into the 2B* ORF at late timepoints, frameshifting temporally regulates the expression ratio of structural and enzymatic proteins [7]. In that work, we also showed that frameshifting in EMCV is stimulated by the viral 2A protein binding to an RNA stem-loop separated from the shift site sequence by a 13-nt spacer (cf. 5–9 nt for canonical frameshift stimulators). Thus cellular levels of viral 2A protein provide the temporal switch. Concurrent with the ribosome profiling analysis, we also assessed frameshifting efficiency via metabolic labelling of viral protein products. While potentially less accurate and less sensitive at early timepoints than ribosome profiling, metabolic labelling allowed assessment of a greater number of mutants at late timepoints, and provided an independent assessment of frameshifting efficiency in the context of virus infection. Here we present the results of the metabolic labelling experiments which could not be accommodated within [7]. Confirming previous results, we show that frameshifting depends on the G_GUU_UUU shift site sequence and the 3′-adjacent stem-loop structure. We also show that frameshifting is not appreciably affected by the ‘StopGo’ sequence present just upstream of the frameshift site.

We used previously generated wild-type (WT), SS and LV-WT mutant viruses (Fig. 1b) [7] which were based on the EMCV subtype mengovirus cDNA, pMC0 of [8]. In the SS mutant, the WT frameshift site G_GUU_UUU is mutated to A_GUG_UUU. These mutations do not alter the polyprotein amino acid sequence but are expected to inhibit PRF. The LV-WT mutant, in which the shift site is WT but StopGo is inhibited by mutating the NPGP sequence to NPLV, was used to assess whether StopGo affects PRF. We also generated tagged versions of these viruses (V5-WT, V5-SS, LV-V5-WT), in which a sequence encoding a V5 tag and glycine-serine linker (GKPIPNPLLGLDSTGSGSGS encoded by GGC AAG CCT ATC CCT AAC CCT CTC TTG GGA CTC GAT TCT ACA GGA TCT GGC TCC GGC AGC) was inserted directly after the final proline codon of the StopGo sequence. Finally, we generated a tagged virus with both StopGo and the shift site mutated (LV-V5-SS). Cells were transfected with T7 transcripts and virus recovered as described previously [7]. All viruses were able to replicate in cell culture.

To confirm inhibition of PRF by the SS mutations and inhibition of StopGo co-translational separation by the LV mutations, we performed Western blot analysis of 2A and 2B* expression in the V5-tagged viruses using polyclonal rabbit antibodies anti-2B*(N-term) and anti-2B*(C-term), raised against the N-terminal 12 aa shared by 2B and 2B* and the C-terminal 14 aa of 2B*, respectively, and mouse monoclonal antibody Mu-anti-2A, raised against the 2A peptide HKRIRPFRLP. BHK-21 or L929 cells were infected with V5-tagged WT or mutant viruses at m.o.i.~10 and lysates were prepared at 8 h p.i.

Mu-anti-2A detected a product migrating just above 15 kDa for V5-WT and V5-SS viruses (Fig. 1c, right, lanes 1–2). This product is presumably 2A (16.7 kDa) and, as expected, it did not react with any of the other antibodies. For LV-V5-WT virus, inhibition of StopGo is expected to fuse 2A to downstream products. Mu-anti-2A detected two products, migrating at ~20 and ~34 kDa (Fig. 1c, right, lane 3). Both products were also detected by anti-V5 (Fig. 1c, left, lane 3) and anti-2B*(N-term) (Fig. 1d, right, lane 3). The ~34 kDa product was not detected for LV-V5-SS virus (Fig. 1c, d, lane 4) indicating that it is a frameshift product, presumably V5-tagged 2A-2B* (32.8 kDa). The ~20 kDa product was detected for both LV-V5-WT and LV-V5-SS viruses, indicating that it is a zero-frame product. Consistent with this, only the ~34 kDa product was detected with anti-2B*(C-term) (Fig. 1e, right, lane 3). The ~20 kDa product is too small to be V5-tagged 2A-2B (35.1 kDa), and is presumed to result from proteolytic cleavage by the virus 3C protease between phylogenetically conserved Q and G residues just three amino acids downstream of the shift site, giving rise to 2A fused to the V5-tagged N-terminal 15 amino acids of 2B (20.5 kDa; referred to here as 2A-V5-2B^N^) [4, 6, 9]. The ~20 and ~34 kDa products were also detected for viruses with an intact StopGo sequence, but at a much lower level (Fig. 1c, d, lane 1). These products arise because StopGo-mediated co-translational separation of 2A and 2B/2B* is not 100 % efficient. Anti-2B*(C-term) also detected a product, migrating at ~19 kDa for WT-V5 virus (Fig. 1e, right, lane 1). This product was not detected for the shift site (SS) or StopGo (LV) mutant viruses and is presumably V5-tagged 2B* (16.0 kDa). The product is also detected, for WT-V5 virus, by anti-2B*(N-term) and anti-V5 (Fig. 1d, lane 1). V5-tagged 2B (18.3 kDa) was not reliably detected with anti-V5 or anti-2B*(N-term) (which should recognize the N-terminal 12 aa common to both 2B and 2B*). We also failed to observe V5-tagged 2A-2B (35.1 kDa) in the StopGo (LV) mutants. The failure to observe full-length 2B or 2A-2B suggests that 2B may be efficiently cleaved at the aforementioned 3C cleavage site (producing 2B′ in Fig. 1f). Note however that neither 2B nor 2B′ were reliably identified by radiolabelling (below), perhaps due to co-migration with other products. A product migrating at ~29 kDa, detected with Mu-anti-2A, anti-V5 and anti-2B*(N-term) but not anti-2B*(C-term), for LV-V5-WT virus only, likely represents a C-terminally truncated version of 2A-V5-2B*, potentially arising from proteolytic cleavage within 2B*.

**Fig. 2.**
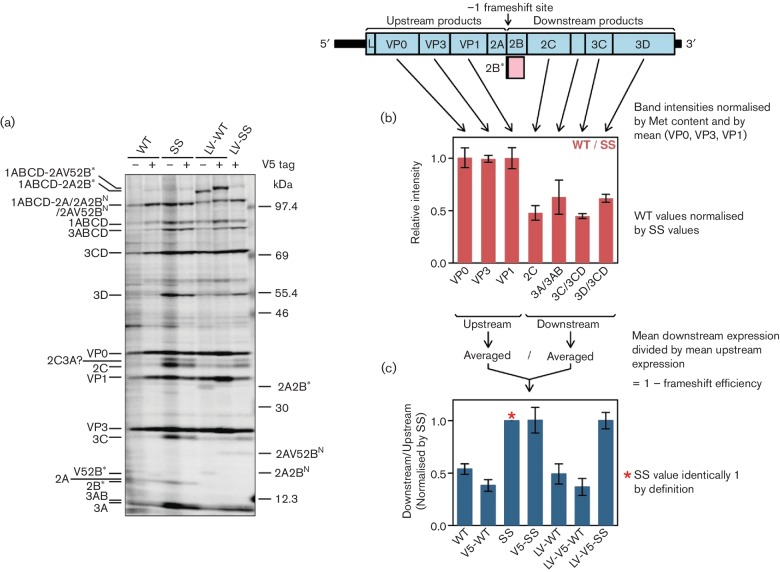
EMCV frameshifting efficiencies measured by metabolic labelling. (a) Phosphorimager image of SDS-PAGE of lysates of L929 cells infected with WT or mutant viruses. Positions of EMCV proteins are indicated. (b) Ratio of band intensities between WT and SS mutant viruses. (c) Mean ratio of measurable polyprotein products encoded downstream of the frameshift site to products encoded upstream of the frameshift site, normalized by the SS mutant. Values in (b, c) show means±sd of three biological replicates.

To calculate PRF efficiences in the context of virus infection, L929 cells were infected at an m.o.i. of ~10 and translation products were radiolabelled from 9 to 10 h p.i. with [^35^S] methionine, separated by 6–15 % SDS-PAGE (Fig. 2a), and radioactivity in virus-specific products was quantified by phosphorimager as described previously [7]. EMCV infection results in efficient shut-off of host cell protein synthesis via inhibition by the viral L and 2A proteins of active nucleocytoplasmic trafficking and cap-dependent translation [10, 11]; thus most well-expressed radiolabelled products correspond to virus proteins. The intensity for each WT virus product was measured, normalized by methionine content, and then by the mean value for VP0, VP3 and VP1 to control for lane loading. Next, to factor out differences in protein turnover besides unquantified processing intermediates, for each biological replicate the WT values for VP0, VP3, VP1, 2C, 3A + 3AB, 3C + 3CD and 3D + 3CD were normalized by corresponding values for SS mutant virus (Fig. 2b). Then the normalized values for 2C, 3A + 3AB, 3C + 3CD and 3D + 3CD (i.e. products encoded downstream of the frameshift site) were averaged and divided by the average of the values for VP0, VP3 and VP1 (i.e. products encoded upstream of the frameshift site). This gives an estimate of the fraction of ribosomes that escape a −1 PRF (Fig. 2c); one minus this value estimates the PRF efficiency. The calculation uses the most easily measurable discrete virus protein bands while some unprocessed or partially processed polyprotein products were ignored, though normalization by the SS mutant is expected to largely correct for these omissions. We repeated this procedure also for the LV-WT mutant and the V5-tagged viruses. The mean PRF efficiencies calculated for WT, LV-WT, V5-WT and LV-V5-WT viruses were in the range 46–63 % while V5-SS and LV-V5-SS had protein expression patterns similar to SS (Fig. 2c).

**Fig. 3.**
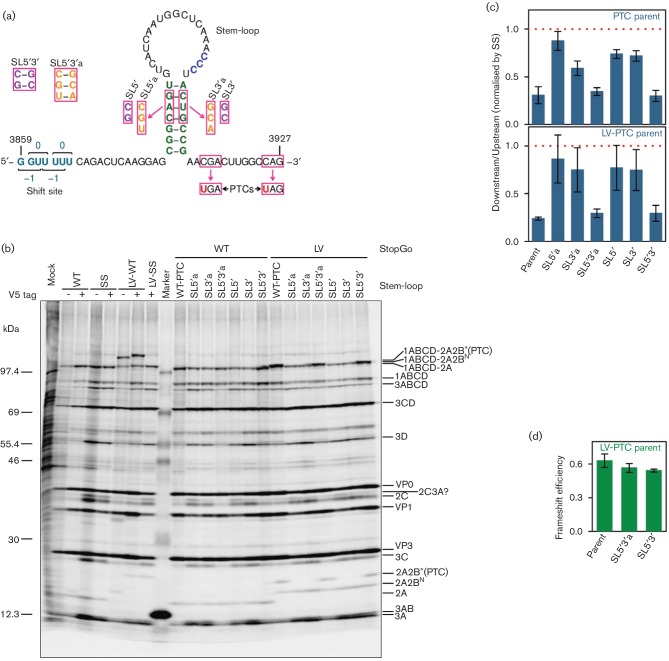
Role of a 3′ stem-loop structure in frameshift stimulation. (a) Mutations introduced to destabilize the predicted stem-loop structure (SL5′, SL3′, SL5′a, SL3′a) or restore it with altered base-pairings (SL5′3′, SL5′3′a). Mutated nucleotides are indicated with pink boxes. Mutants were generated in the context of a parent virus, WT-PTC, in which the 2B* ORF was truncated via the introduction of two PTCs. NPGP to NPLV StopGo mutant versions of the six stem-loop mutants and the WT-PTC parent were also constructed. (b) Phosphorimager image of SDS-PAGE of lysates of L929 cells infected with WT or mutant viruses. The positions of EMCV proteins are shown on the right. Note that Fig. 2(a) is identical to the left-hand portion of this figure, reproduced here for clarity. (c) Mean ratio of measurable polyprotein products encoded downstream of the frameshift site to products encoded upstream of the frameshift site, normalized by SS mutant virus. The red dotted line at 1 indicates the SS mutant ‘no frameshift’ baseline. (d) Direct PRF efficiency estimates based on 2A-2B*(PTC) and 2A-2B^N^ expression in the three LV PTC mutants with a quantifiable 2A-2B*(PTC) band. Values in (c, d) show means±sd of three biological replicates.

In previous work, a downstream stem-loop structure, separated from the frameshift site by a 13-nt ‘spacer’, was identified bioinformatically and confirmed experimentally [5, 7]. This positioning is inconsistent with canonical mRNA structure stimulators of −1 PRF, which are separated from the shift site by just 5–9 nt. To assess the PRF-stimulatory role of the stem-loop in the context of the virus genome during infection, we made several new mutant EMCVs. In SL5′ we altered the 5′ arm of the stem; in SL3′ we altered the 3′ arm of the stem; and in SL5′3′ we combined both mutations to restore the predicted RNA structure (Fig. 3a). These mutations are non-synonymous with regards to the 2B and 2B* amino acid sequences, but are present in some natural EMCV isolates (e.g. GenBank accession KC310737) and, indeed, all three viruses were found to replicate in cell culture. We also prepared another set of SL mutants (SL5′a, SL3′a, SL5′3′a; Fig. 3a) with an additional base-pair change. To rule out possible effects of the amino acid changes in 2B* influencing virus replication, all stem-loop mutations were generated in the context of a parent virus, WT-PTC, in which two premature termination codons (PTCs) were introduced into the 2B* reading frame without affecting the polyprotein amino acid sequence (Fig. 3a).

The effect of these mutations on PRF was assessed by metabolic labelling (Fig. 3b, lanes 10–16). Mutants in which the stem-loop was disrupted (SL5′, SL3′, SL5′a, SL3′a) displayed a pattern of virus protein expression similar to that of SS mutant virus, while the stem-loop restoration mutants (SL5′3′ and SL5′3′a) displayed a pattern of virus protein expression similar to that of WT and WT-PTC viruses. PRF efficiencies were estimated as above based on the ratio of expression of products encoded downstream and upstream of the shift site, normalized by SS mutant virus. The ratio of downstream to upstream products for the SL mutants was substantially greater than for WT virus, indicating significant, but probably not complete, disruption of PRF (Fig. 3c).

We also constructed StopGo-inhibited (LV mutant) versions of all the SL mutant EMCVs so that we could directly observe the PRF product 2A-2B*(PTC) [where ‘2B*(PTC)’ represents the C-terminally truncated 2B* as a result of the PTC mutation in the parent]. 2B*(PTC) itself (3.2 kDa) was too small to see by SDS-PAGE, but when fused to 2A (19.9 kDa total) it could be visualized. The LV viruses (Fig. 3b, lanes 17–23) had ratios of downstream to upstream products similar to the corresponding non-LV viruses (Fig. 3c). Moreover, 2A-2B*(PTC) was only apparent in WT-PTC and the stem-loop restoration mutants, SL5′3′ and SL5′3′a (Fig. 3b, lanes 17, 20, 23). For these three viruses, PRF efficiencies were also estimated by quantifying the radioactivity in 2A-2B*(PTC) (frameshift product) and 2A-2B^N^ (i.e. 2A fused to the N-terminus of 2B), normalizing by methionine content, and taking the ratio [2A-2B*(PTC)]/([2A-2B^N^]+[2A-2B*(PTC)]). This calculation assumes 100 %-efficient cleavage of 2A-2B in the LV mutants at the Q|G 3C-protease cleavage site near the N-terminus of 2B (2B residues 15–16) as supported by Western analysis (Fig. 1). Using this method, the PRF efficiencies for these three viruses had values in the range 54–63 % (Fig. 3d).

Previously we tested WT and five mutants (SS, WT-SL, SS-SL, LV-WT and LV-SS-SL; where SL indicates three synonymous mutations within the stem-loop) in the context of the full-length viral genome during virus infection using ribosome profiling [7]. Here we test WT and a total of 20 virus mutants by metabolic labelling, of which only SS and LV-WT overlap with the previously tested mutants. The mean PRF efficiencies for WT, V5-WT, LV-WT, LV-V5-WT, WT-PTC, SL5′3′, SL5′3′a, LV-WT-PTC, LV-SL5′3′ and LV-SL5′3′a fall in the range 46–76 % (64±9 %, mean±sd), while direct measurement of 2A-2B*(PTC) in the latter three gave values 63, 57 and 54 %. These values support efficient frameshifting at 9–10 h p.i. but are consistently lower than those measured previously using ribosome profiling (69 , 70 and 62 % at 8 h p.i. for, respectively, two WT biological repeats and LV-WT) [7]. The ribosome profiling analysis is expected to be significantly more accurate. Interestingly, measurements in the related Theiler’s murine encephalomyelitis virus (TMEV) using radiolabelling put the TMEV PRF efficiency at 74–82 % at 6–7 h p.i. [6]. Despite the modest discrepancy in the absolute level of late timepoint frameshifting, the current results show that frameshifting in EMCV does not depend on the StopGo sequence (cf. WT with LV-WT; Fig. 2c) but does depend on the stem-loop structure (cf. parent, SL5′3′ and SL5′3′a with SL5′, SL3′, SL5′a and SL3′a; Fig. 3c), thus supporting the results of [7] with a larger number of mutants in the context of the virus genome during infection. The results are also consistent with a mutational analysis of the stem-loop in cell-free translation systems (rabbit reticulocyte lysate and wheat germ extract), where frameshifting could be recapitulated to a level of ~20 % (instead of the ~70 % seen in virus-infected cells) upon addition of recombinant 2A [7].
